# Nucleolar Protein Trafficking in Response to HIV-1 Tat: Rewiring the Nucleolus

**DOI:** 10.1371/journal.pone.0048702

**Published:** 2012-11-15

**Authors:** Mohamed Ali Jarboui, Carlo Bidoia, Elena Woods, Barbara Roe, Kieran Wynne, Giuliano Elia, William W. Hall, Virginie W. Gautier

**Affiliations:** 1 Centre for Research in Infectious Diseases (CRID), School of Medicine and Medical Science (SMMS), University College Dublin (UCD), Dublin, Ireland; 2 Mass Spectrometry Resource (MSR), Conway Institute for Biomolecular and Biomedical Research, University College Dublin (UCD), Dublin, Ireland; Ghent University, Belgium

## Abstract

The trans-activator Tat protein is a viral regulatory protein essential for HIV-1 replication. Tat trafficks to the nucleoplasm and the nucleolus. The nucleolus, a highly dynamic and structured membrane-less sub-nuclear compartment, is the site of rRNA and ribosome biogenesis and is involved in numerous cellular functions including transcriptional regulation, cell cycle control and viral infection. Importantly, transient nucleolar trafficking of both Tat and HIV-1 viral transcripts are critical in HIV-1 replication, however, the role(s) of the nucleolus in HIV-1 replication remains unclear. To better understand how the interaction of Tat with the nucleolar machinery contributes to HIV-1 pathogenesis, we investigated the quantitative changes in the composition of the nucleolar proteome of Jurkat T-cells stably expressing HIV-1 Tat fused to a TAP tag. Using an organellar proteomic approach based on mass spectrometry, coupled with Stable Isotope Labelling in Cell culture (SILAC), we quantified 520 proteins, including 49 proteins showing significant changes in abundance in Jurkat T-cell nucleolus upon Tat expression. Numerous proteins exhibiting a fold change were well characterised Tat interactors and/or known to be critical for HIV-1 replication. This suggests that the spatial control and subcellular compartimentaliation of these cellular cofactors by Tat provide an additional layer of control for regulating cellular machinery involved in HIV-1 pathogenesis. Pathway analysis and network reconstruction revealed that Tat expression specifically resulted in the nucleolar enrichment of proteins collectively participating in ribosomal biogenesis, protein homeostasis, metabolic pathways including glycolytic, pentose phosphate, nucleotides and amino acids biosynthetic pathways, stress response, T-cell signaling pathways and genome integrity. We present here the first differential profiling of the nucleolar proteome of T-cells expressing HIV-1 Tat. We discuss how these proteins collectively participate in interconnected networks converging to adapt the nucleolus dynamic activities, which favor host biosynthetic activities and may contribute to create a cellular environment supporting robust HIV-1 production.

## Introduction

The nucleolus is a highly ordered subnuclear compartment organised around genetic loci called nucleolar-organising regions (NORs) formed by clusters of hundreds of rDNA gene repeats organised in tandem head-to-tail repeat [1], [2]. A membrane-less organelle originally described as the “Ribosome Factory”, the nucleolus is dedicated to RNA-polymerase-I-directed rDNA transcription, rRNA processing mediated by small nucleolar ribonucleoproteins (soRNPs) and ribosome assembly. Ribosome biogenesis is essential for protein synthesis and cell viability [2] and ultimately results in the separate large (60S) and small (40S) ribosomal subunits, which are subsequently exported to the cytoplasm. This fundamental cellular process, to which the cell dedicates most of its energy resources, is tightly regulated to match dynamic changes in cell proliferation, growth rate and metabolic activities [3].

The nucleolus is the site of additional RNA processing, including mRNA export and degradation, the maturation of uridine-rich small nuclear RNPs (U snRNPs), which form the core of the spliceosome, biogenesis of t-RNA and microRNAs (miRNAs) [4]. The nucleolus is also involved in other cellular processes including cell cycle control, oncogenic processes, cellular stress responses and translation [4].

The concept of a multifunctional and highly dynamic nucleolus has been substantiated by several studies combining organellar proteomic approaches and quantitative mass spectrometry, and describing thousands of proteins transiting through the nucleolus in response to various metabolic conditions, stress and cellular environments [5], [6], [7], [8], [9], [10], [11], [12], [13], [14], [15], [16]. Collectively, the aforementioned studies represent landmarks in understanding the functional complexity of the nucleolus, and demonstrated that nucleolar proteins are in continuous exchange with other nuclear and cellular compartments in response to specific cellular conditions.

Of importance, the nucleolus is also the target of viruses including HIV-1, hCMV, HSV and KSHV, as part of their replication strategy [2], [17]. Proteomics studies analysing the nucleoli of cells infected with Human respiratory syncytial virus (HRSV), influenza A virus, avian coronavirus infectious bronchitis virus (IBV) or adenovirus highlighted how viruses can distinctively disrupt the distribution of nucleolar proteins [2], [17], [18], [19], [20], [21], [22], [23], [24]. Interestingly, both HIV-1 regulatory proteins Tat and Rev localise to the nucleoplasm and nucleolus. Both their sequences encompass a nucleolar localisation signal (NoLS) overlapping with their nuclear localisation signal (NLS), which governs their nucleolar localisation [25], [26], [27], [28], [29], [30], [31]. Furthermore, Tat and Rev interact with the nucleolar antigen B23, which is essential for their nucleolar localisation [25], [26], [27], [28], [29], [30]. Nevertheless, a recent study described that in contrast to Jurkat T-cells and other transformed cell lines where Tat is associated with the nucleus and nucleolus, in primary T-cells Tat primarily accumulates at the plasma membrane, while trafficking via the nucleus where it functions [32]. While the regulation of their active nuclear import and/or export, as mediated by the karyopherin/importin family have been well described, the mechanisms distributing Tat and Rev between the cytoplasm, nucleoplasm and the nucleolus remains elusive [33], [34], [35], [36], [37], [38], [39], [40], [41], [42], [43], [44], [45], [46], [47], [48]. Importantly, two major studies by Machienzi *et al.* have revealed important functional links between HIV-1 replication and the nucleolus [49], [50]. First, they could inhibit HIV-1 replication and Tat transactivation function employing a TAR decoy specifically directed to the nucleolus. Furthermore, using a similar approach, with an anti-HIV-1 hammerhead ribozyme fused to the U16 small nucleolar RNA and therefore targeted to the nucleolus, they could dramatically suppress HIV-1 replication. Collectively, these findings strongly suggest that HIV-1 transcripts and Tat nucleolar trafficking are critical for HIV-1 replication. However the nature of these contributions remains to be elucidated.

In this report, we systematically analysed the nucleolar proteome perturbations occurring in Jurkat T-cells constitutively expressing HIV-1 Tat, using a quantitative mass spectrometry approach. Following the detailed annotation of the quantitative abundance changes in the nucleolar protein composition upon Tat expression, we focussed on the Tat-affected cellular complexes and signalling pathways associated with ribosome biogenesis, spliceosome, molecular chaperones, DNA replication and repair and metabolism and discuss their potential involvement in HIV-1 pathogenesis.

## Results

### 1 Experimental Design

**Figure 1:**
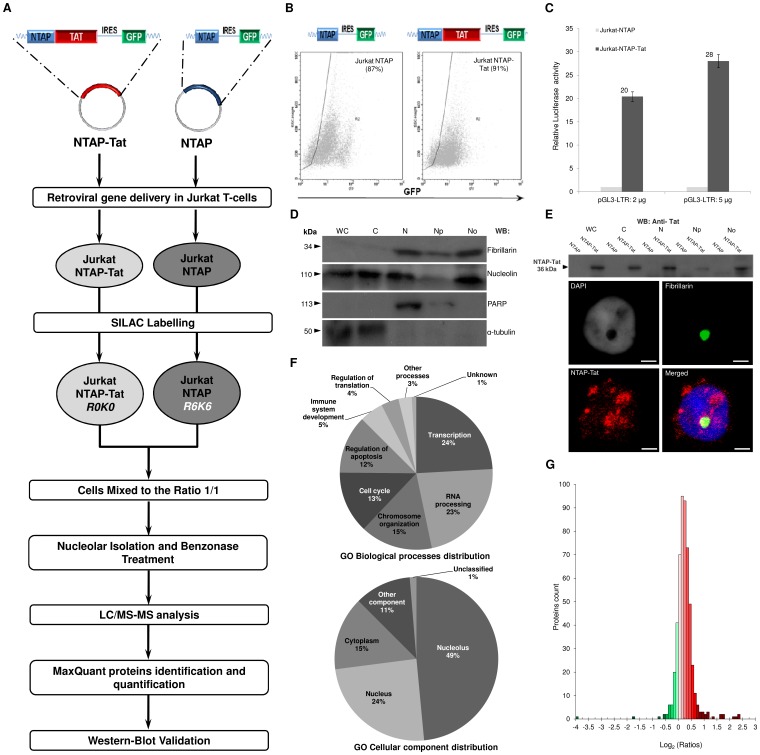
Proteomic workflow and validation studies of the SILAC analysis of Jurkat-T cells expressing HIV-1 Tat. **A.** Organellar proteomic workflow for the analysis of quantitative changes in Jurkat NTAP-Tat versus the Jurkat NTAP cells. **B.** Designed cassettes for NTAP and NTAP-Tat. GFP is cloned downstream the NTAP-Tat and NTAP genes and its translation is independently controlled by IRES. FACS analysis of the Jurkat NTAP-Tat and Jurkat NTAP showing a highly enriched population of polyclonal transduced cells. **C.** We transfected the Jurkat NTAP-Tat and Jurkat NTAP using 2 and 5 µg of pGL3-LTR plasmid. HIV-1 LTR luciferase reporter gene assay confirmed that the NTAP-Tat is functionally active. **D.** Validation of the subcellular fractionation from mixed Jurkat NTAP-Tat (R0K0) and Jurkat NTAP (R6K6) (1∶1). Each fraction (10 µg of protein per lane) was checked by Western-Blot using anti-nucleolin (C23), anti-fibrillarin, anti-α-tubulin and anti-PARP antibodies. The molecular weight (kDa) of each protein is indicated on the left. (Fractions: WC: whole cells, C: Cytoplasmic, N: Nuclear, Np: Nucleoplasmic and No: Nucleolar). **E.** Expression and subcellular distribution of NTAP-Tat in Jurkat T-cells using Western-Blot analysis. NTAP-Tat (36 kDa) was detected using anti-HIV-1 Tat antibody (ab43014, Abcam). For immunofluorescence analysis, Jurkat NTAP-Tat cells were stained for fibrillarin (green), HIV-1 Tat (red), and DAPI (Blue or in grey contrast on the top left panel). NTAP-Tat overlaps with fibrillarin in the nucleolar compartment. (Bar: 2 µm). **F.** GO biological processes and cellular components distribution of the nucleolar proteome of mixed cells. **G.** Distribution of the changes in protein abundance in the nucleolus of Jurkat T-cells upon HIV-1 Tat expression. Relative abundance is plotted as Log2 (SILAC ratios). Green denotes depletion, while red denotes enrichment.

In this study, we investigated the quantitative changes in the nucleolar proteome of Jurkat T cells constitutively expressing HIV-1 Tat (86aa) versus their Tat-negative counterpart, using stable isotope labelling with amino acids in cell culture (SILAC) technology, followed by ESI tandem mass spectrometry and implemented the experimental approach described in Figure 1A. First, using retroviral gene delivery, we transduced HIV-1 Tat fused to a tandem affinity purification (TAP) tag (consisting of two protein G and a streptavidin binding peptide) or TAP tag alone (control vector) in Jurkat leukemia T cell clone E6-1 and sorted the transduced cells (GFP positive) by FACS. This resulted in a highly enriched population of polyclonal transduced cells presenting different expression levels of the transgene (Figure 1B). The functionality of TAP-Tat was confirmed by transfecting Jurkat TAP-Tat and TAP cells with a luciferase reporter gene vector under the control of the HIV-1 LTR (pGL3- LTR) [36]. TAP-Tat up regulated gene expression from the HIV-1 LTR by up to 28 fold compared to control (Figure 1C). To further address the functionality of Tat fused to TAP, we compared Jurkat TAP-Tat with Jurkat-tat, a cell line stably expressing untagged Tat [51]. Both cell line exhibited comparable HIV-1 LTR activity following transfection with pGL3- LTR (Figure S1). Next, Tat expression and subcellular localization was verified by subcellular fractionation followed by WB analysis (Figure 1E). TAP-Tat displayed a prominent nuclear/nucleolar localization but could also be detected in the cytoplasm. These observations were further validated by immunofluorescence microscopy (Figure 1E). Of note, Jurkat-tat presented similar patterns for Tat subcellular distribution as shown by immunofluorescence microscopy and subcellular fractionation followed by WB analysis (Figure S2 and S3). We next compared the growth rate and proliferation of the Jurkat TAP and TAP-Tat cell lines (Materials and Methods S1), which were equivalent (Figure S4A). Similarly, FACS analysis confirmed that the relative populations in G1, S, and G2/M were similar for Jurkat TAP-Tat and TAP cells (Figure S4B).

We labeled Jurkat TAP-Tat and Jurkat TAP cells with light (R0K0) and heavy (R6K6) isotope containing arginine and lysine, respectively. Following five passages in their respective SILAC medium, 85 million cells from each culture were harvested, pooled and their nucleoli were isolated as previously described (Figure 1A) [52]. Each step of the procedure was closely monitored by microscopic examination. To assess the quality of our fractionation procedure, specific enrichment of known nucleolar antigens was investigated by Western Blot analysis (Figure 1D). Nucleolin (110 kDa) and Fibrillarin (FBL) (34 kDa), two major nucleolar proteins known to localise to the granular component of the nucleolus, were found to be highly enriched in the mixed nucleolar fraction. Of note, nucleolin was equally distributed between the nuclear and cytoplasmic fractions. This distribution pattern for nucleolin appears to be specific for Jurkat T-cells as show previously [52], [53]. The nuclear protein PARP-1 (Poly ADP-ribose polymerase 1) (113 kDa) was present in the nuclear and nucleoplasmic fraction but was depleted in the nucleolar fraction. Alpha-tubulin (50 kDa) was highly abundant in the cytoplasmic fraction and weakly detected in the nuclear fractions. Collectively, these results confirmed that our methods produced a highly enriched nucleolar fraction without significant cross contamination.

Subsequently, the nucleolar protein mixture was trypsin-digested and the resulting peptides were analysed by mass spectrometry. Comparative quantitative proteomic analysis was performed using MaxQuant to analyse the ratios in isotopes for each peptide identified. A total of 2427 peptides were quantified, representing 520 quantified nucleolar proteins. The fully annotated list of the quantified nucleolar proteins is available in Table S1 and the raw data from the mass spectrometry analysis was deposited in the Tranche repository database (https://proteomecommons.org/tranche/), which can be accessed using the hash keys described in materials and methods. We annotated the quantified proteins using the ToppGene Suite tools [54] and extracted Gene Ontology (GO) and InterPro annotations [55]. The analysis of GO biological processes (Figure 1F) revealed that the best-represented biological processes included transcription (24%), RNA processing (23%), cell cycle process (13%) and chromosome organisation (15%), which reflects nucleolar associated functions and is comparable to our previous characterisation of Jurkat T-cell nucleolar proteome [52]. Subcellular distribution analysis (Figure 1F) revealed that our dataset contained proteins known to localise in the nucleolus (49%), in the nucleus (24%) while 15% of proteins were previously described to reside exclusively in the cytoplasm. The subcellular distribution was similar to our previous analysis of the Jurkat T-cell nucleolar proteome [52].

### 2 Quantitative Proteomic Profiling of the Nucleoli of Jurkat TAP-Tat versus Jurkat TAP

The changes in relative protein abundance in the nucleolus of T-cells associated with HIV-1 Tat expression are listed and annotated in Table S1. The distribution of protein ratios are represented in Figure 1G as log_2_ (abundance change). The SILAC ratios indicate changes in protein abundance in the nucleolar fraction of Jurkat TAP-Tat cells in comparison with Jurkat TAP cells. The distribution of the quantified proteins followed a Gaussian distribution (Figure 1G). A total of 49 nucleolar proteins exhibited a 1.5 fold or greater significant change (p<0.05) upon Tat expression (Table 1). Of these, 30 proteins were enriched, whereas 19 proteins were depleted. Cells displayed no changes in the steady state content of some of the major and abundant constituents of the nucleolus, including nucleophosmin (NPM1/B23), C23, FBL, nucleolar protein P120 (NOL1), and nucleolar protein 5A (NOL5A). The distinct ratios of protein changes upon Tat expression could reflect specific nucleolar reorganization and altered activities of the nucleolus.

**Table 1:** List of proteins showing a significant change in abundance in the nucleoli of Jurkat T-cells following expression of HIV-1 Tat.

Weigheted Ratios H/L	Gene Symbol	Protein Names
5.26	NHP2L1	NHP2 non-histone chromosome protein 2-like 1
5.25	RPL14	60S ribosomal protein L14
4.75	LDHB	Lactate Dehydrogenase b
4.44	HSP90AB1	Heat shock 90 kD protein, beta a
3.40	PFKP	6-phosphofructokinase type C
3.30	MCM7	Minichromosome maintenance complex component 7
3.09	HNRNPA3	Heterogeneous nuclear ribonucleoprotein A3
3.04	RPL17	60S ribosomal protein L17
2.58	FUSIP1	FUS-interacting serine-arginine-rich protein 1
2.26	FKBP4	FK506-binding protein 4
2.16	ARL8B	ADP-ribosylation factor-like protein 8B
2.15	RPL27	60S ribosomal protein L27
2.11	G6PD	Glucose-6-phosphate 1-dehydrogenase
2.03	RPS2	40S ribosomal protein S2
1.90	PSAT1	Phosphoserine aminotransferase 1
1.88	STAT3	Signal transducer and activator of transcription 3
1.87	HSD17B10	17-beta-hydroxysteroid dehydrogenase 10
1.84	STIP1	Stress-induced-phosphoprotein 1
1.82	CANX	Calnexin
1.75	RPL13	60S ribosomal protein L13
1.74	CTPS	CTP synthase 1
1.70	HINT1	histidine triad nucleotide binding protein 1
1.66	DPYSL2	Dihydropyrimidinase-like 2
1.66	IMPDH2	IMP (inosine 5′-monophosphate) dehydrogenase 2
1.63	MYH9	Myosin heavy chain, type A
1.63	GNB1	Guanine nucleotide-binding protein G subunit beta-1
1.59	CCT6A	T-complex protein 1 subunit 6A (zeta)
1.59	PKM2	Pyruvate kinase muscle 2
1.57	PGK1	phosphoglycerate kinase 1
1.57	HSP90AB2P	Heat shock protein 90-beta b
Weigheted Ratios H/L	Gene Symbol	Protein Names
0.86	TMED10	Transmembrane emp24 domain-containing protein 10
0.84	HNRNPA2B1	Heterogeneous nuclear ribonucleoproteins A2/B1
0.84	CDK6	Cell division protein kinase 6
0.84	GPR133	G-protein coupled receptor 133
0.82	EZR	ezrin
0.82	CASP10	Caspase 10
0.81	SNRPA	U1 small nuclear ribonucleoprotein A
0.81	BOP1	Block of proliferation 1 protein
0.78	ADAR	adenosine deaminase, RNA-specific
0.77	MAGOHB	Protein mago nashi homolog 2
0.76	SUMO3	Small ubiquitin-related modifier 3
0.76	UTP15	U3 small nucleolar RNA-associated protein 15 homolog
0.74	KIF2A	Kinesin-2
0.72	PSMD13	26S proteasome non-ATPase regulatory subunit 13
0.69	RFC4	Replication factor C subunit 4
0.68	YWHAG	14-3-3 protein gamma
0.60	DBN1	Drebrin
0.29	RPL35A	60S ribosomal protein L35a
0.06	USP20	Ubiquitin specific peptidase 20

Ratios over one, correspond to proteins with an increase in abundance, while ration less than one correspond to proteins with a decreased abundance. Significance B (p value<0.05).

**Figure 2:**
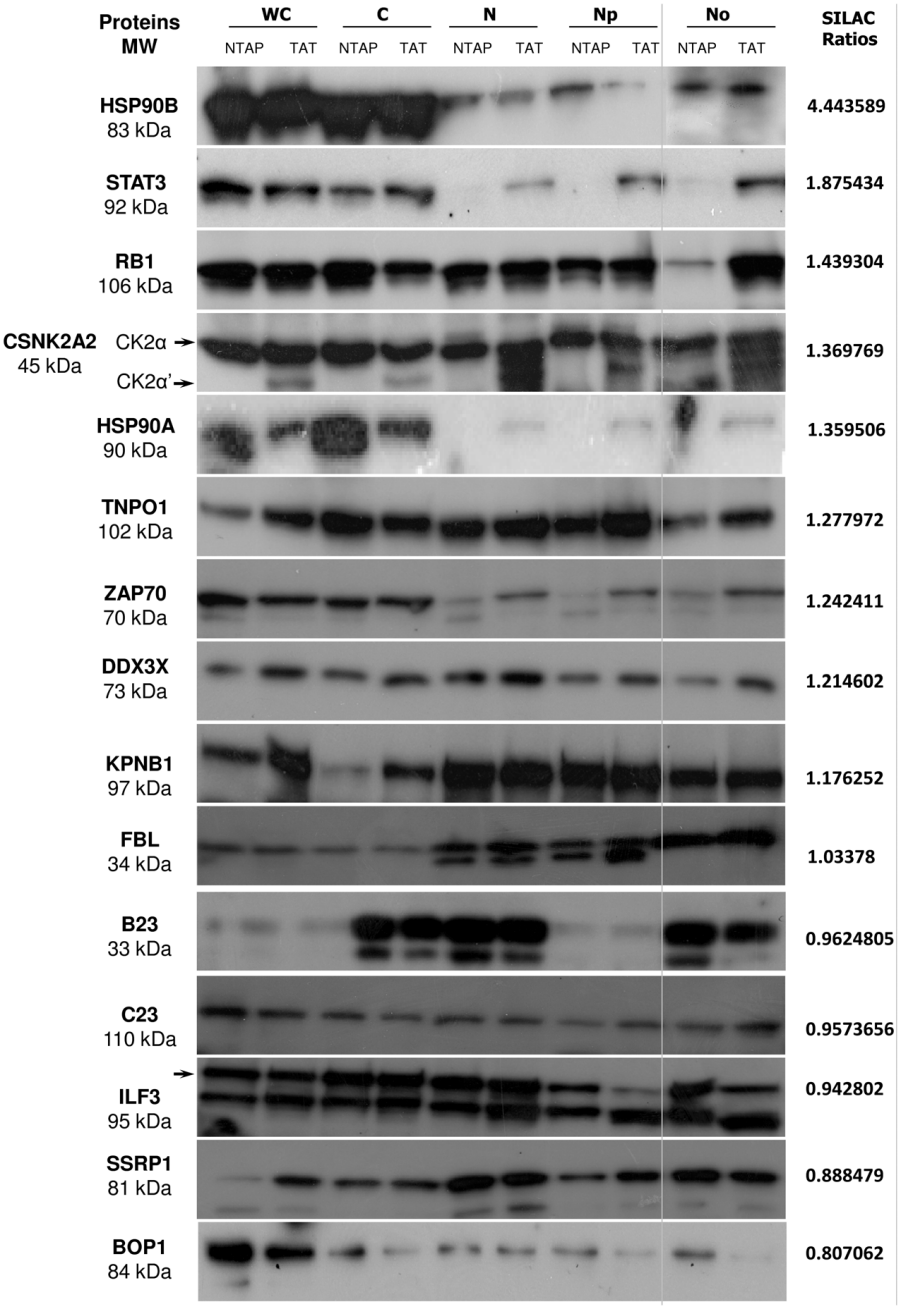
Western-Blot validation of the SILAC quantitative analysis. Western-Blot results comparing expression levels of selected 15 proteins to their SILAC ratios (right panel). 10 µg total protein of each subcellular fraction (WC: whole cells, C: Cytoplasmic, N: Nuclear, Np: Nucleoplasmic and No: Nucleolar) from Jurkat NTAP (NTAP) and Jurkat NTAP-Tat (TAT) were resolved by SDS-PAGE, blotted and probed with indicated antibodies. For HSP90B, as the nucleolar detection level was low, we increased separately the amount of total protein loaded to 20 µg.

We performed WB analysis to validate the SILAC-based results obtained by our quantitative proteomic approach (Figure 2). 15 selected proteins displayed differential intensity in the nucleolar fractions upon Tat expression, including 9 enriched (HSP90β, STAT3, pRb, CK2α, CK2α’, HSP90α, Transportin, ZAP70, DDX3), and 3 depleted (ILF3, BOP1, and SSRP1) proteins. In addition, we also tested by WB analysis, protein abundance not affected by Tat expression (Importin beta, FBL, B23, C23). These results highlight the concordance in the trend of the corresponding SILAC ratios, despite some differences in the quantitative ranges. Of note, using WB, we could observe a change of intensity for protein with a SILAC fold change as low as 1.25-fold.

Of note, the question remains as to which fold change magnitude might constitute a biologically relevant consequence. On the one hand, the threshold of protein abundance changes can be determined statistically and would then highlight the larger abundance changes as illustrated in Table 1. Alternatively, the coordinated enrichment or depletion of a majority of proteins belonging to a distinct cellular complex or pathway would allow the definition of a group of proteins of interest and potential significance. Therefore, we next focused on both enriched or depleted individual proteins with activities associated with HIV-1 or Tat molecular pathogenesis, and on clustered modifications affecting entire cellular signaling pathways and macromolecular complexes.

### 3 Characterisation of Signaling Protein Abundance Changes Associated with HIV-1 Pathogenesis

We initially focused on signaling proteins interacting with Tat and/or associated HIV-1 molecular pathogenesis and whose abundance in the nucleolus was modulated by Tat expression.

#### Phospho-protein phosphatases

Phospho-protein phosphatase PP1 and PP2A are essential serine/threonine phosphatases [56], [57]. Importantly, PP1 accounts for 80% of the Ser/Thr phosphatase activity within the nucleolus. In our study, PP1 was found to be potentially enriched by 1.52-fold in the nucleolus of Jurkat cells expressing Tat, which supports previous studies describing the nuclear and nucleolar targeting of PP1α by HIV-1 Tat and how PP1 upregulates HIV-1 transcription [58], [59], [60], [61], [62]. PP1 γ was also identified as part of the *in vitro* nuclear interactome [63]. Similarly, PPP2CA, the PP2A catalytic subunit (1.29-fold) and its regulatory subunit PP2R1A (1.27-fold) were similarly enriched upon Tat expression. Interestingly, Tat association with the PP2A subunit promoters results in the overexpression and up regulation of PP2A activity in lymphocytes [64], [65]. Furthermore, PP2A contributes to the regulation of HIV-1 transcription and replication [61], [66].

#### Retinoblastoma Protein

The tumour suppressor gene pRb protein displayed a 1.4-fold change in the nucleolus upon Tat expression [67]. Furthermore, WB analysis confirmed the distinct translocation of pRb from the nucleoplasm to the nucleolus by Tat (Figure 2). Depending on the cell type, pRb can be hyperphosphorylated or hypophosphorylated upon Tat expression and can negatively or positively regulate Tat-mediated transcription respectively [68], [69], [70]. Interestingly, the hyperphosphorylation of pRB triggers in its translocation into the nucleolus [71]. Phosphorylation of pRB is also associated with an increase in ribosomal biogenesis and cell growth [72].

#### STAT3

The transcription factor signal transducer and activator of transcription 3 (STAT3) was significantly enriched (1.86-fold) in the nucleolar fraction by Tat constitutive expression. Furthermore, WB analysis indicated that Tat expression could promote the relocalisation of STAT3 from the cytoplasm to the nucleus, with a distinct enrichment in the nucleolus (Figure 2). Interestingly, previous studies have demonstrated Tat-mediated activation of STAT3 signaling, as shown by its phosphorylation status [73]. Interestingly, STAT3 phosphorylation induced dimerisation of the protein followed its translocation to the nucleus [74].

#### YBX1

YBX1, the DNA/RNA binding multifunctional protein was enriched by 1.38-fold in the nucleolus of Jurkat cells upon Tat expression. Interestingly, YBX1 interacts with Tat and TAR and modulates HIV-1 gene expression [63], [75].

#### ZAP70

The protein tyrosine kinase ZAP70 (Zeta-chain-associated protein kinase 70) was enriched by 1.24-fold in the nucleolus of Jurkat cells expressing Tat [76]. Furthermore, WB analysis revealed that Tat expression could promote the relocalisation of ZAP70 from the cytoplasm to the nucleus, with a distinct enrichment in the nucleolus (Figure 2). Of note, ZAP70 is part of the *in vitro* nuclear Tat interactome [63].

#### Matrin 3

The inner nuclear matrix protein, Matrin 3 (MATR3), presented a 1.39-fold change in the nucleolus of Jurkat cells expressing Tat. It localizes in the nucleolasm with a diffuse pattern excluded from the nucleoli [77]. Matrin 3 has been identified as part of the *in vitro* HIV-1 Tat nuclear interactome [63]. Two recent studies have described Matrin 3 as part of ribonucleoprotein complexes also including HIV-1 Rev and (Rev Response Element) RRE-containing HIV-1 RNA, and promoting HIV-1 post-transcriptional regulation [78], [79], [80].

#### CASP10

The pro-apototic signaling molecule, Caspase 10 (CASP10), was significantly depleted from the nucleolus of Jurkat-Tat cells (0.82-fold) [81]. Importantly, Tat expression down-regulates CASP10 expression and activity in Jurkat cells [82].

#### ADAR1

Adenosine deaminase acting on RNA (ADAR1), which converts adenosines to inosines in double-stranded RNA, was significantly depleted from the nucleolus of Jurkat-Tat cells (0.78-fold). Interestingly, ADAR1 over-expression up-regulates HIV-1 replication via an RNA editing mechanism [83], [84], [85], [86], [87], [88]. Furthermore, ADAR1 belongs to the *in vitro* HIV-1 Tat nuclear interactome [63].

### 4 Rewiring of Nucleolar Protein Interaction Network by HIV-1 Tat

**Figure 3:**
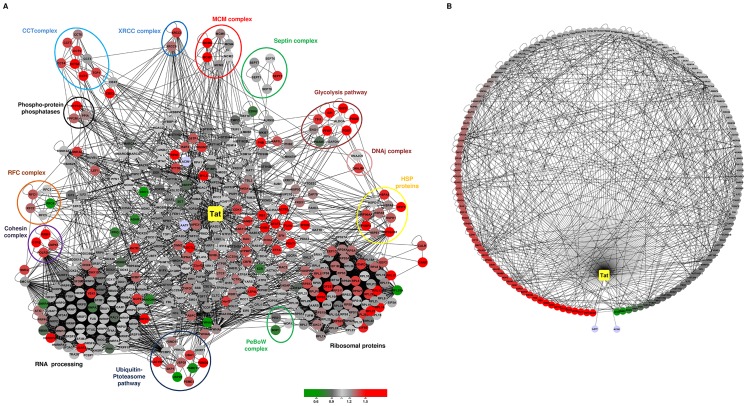
Network analysis of the Tat interactome in nucleoli of Jurkat T-cells. A. The main network contains 416 nodes (proteins) connected with 5060 edges gathered from Protein-Protein interaction databases as described in the text. The central position HIV-1 Tat is highlighted in yellow. **B.** Subnetwork of HIV-1 Tat cellular partners. HIV-1 Tat interacts with 146 proteins out of the 416 proteins identified in the mixed nucleolar fraction. This subnetwork contains 651 edges. Red and green denotes proteins with increased or decreased nucleolar abundance, respectively.

To underline the structural and functional relationships of the nucleolar proteins affected by HIV-1 Tat, we constructed a network representation of our dataset. We employed Cytoscape version 2.6.3 [89] and using the MiMI plugin [90] to map previously characterised interactions, extracted from protein interaction databases (BIND, DIP, HPRD, CCSB, Reactome, IntAct and MINT). This resulted in a highly dense and connected network comprising 416 proteins (nodes) out of the 536 proteins, linked by 5060 undirected interactions (edges) (Figure 3A). Centrality analysis revealed a threshold of 23.7 interactions per protein. Topology analysis using the CentiScaPe plugin [91] showed that the node degree distribution follows a power law (Figure S5), characteristic of a scale-free network. Importantly, when we analysed the clustering coefficient distribution (Figure S6) we found that the network is organised in a hierarchical architecture [92], where connected nodes are part of highly clustered areas maintained by few hubs organised around HIV-1 Tat. Furthermore, node degree connection analysis of our network identified HIV-1 Tat as the most connected protein (Figure S6). Specifically, the topology analysis indicated that the values for Tat centralities were the highest (Node degree, stress, radiality, closeness, betweeness and centroid), characterising Tat as the main hub protein of the nucleolar network. Indeed, a total of 146 proteins have been previously described to interact with Tat (Figure 3B, Table S2). These proteins are involved in a wide range of cellular processes including chromosomal organization, DNA and RNA processing and cell cycle control. Importantly, aver the third of these proteins exhibit an increase in fold ratio change (59 proteins with a ratio >1.2 fold).

### 5 Biomolecular Pathways Dysregulation by HIV-1 Tat

**Figure 4:**
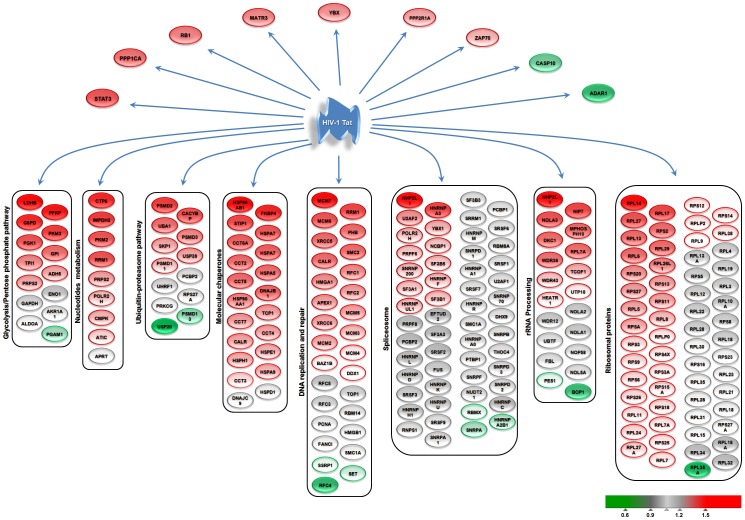
Overall profiles of changes in proteins abundance in the nucleolus of T-cells upon HIV-1 Tat expression. Proteins were organized according GO biological processes and KEGG pathways identified by the ToppGene Suite tools (http://toppgene.cchmc.org/). Red and green denotes proteins with increased or decreased nucleolar abundance, respectively.

In parallel, we characterised the magnitude of the related protein abundance changes observed in distinct cellular pathways (Figure 4).

#### Ribosomal biogenesis

We initially focused on ribosome biogenesis, the primary function of the nucleolus. We could observe a general and coordinated increase in the abundance of ribosomal proteins in the nucleolus by Tat expression (Figure 4). While some ribosomal proteins remained unaffected, Tat caused the nucleolar accumulation of several distinct large and small ribosomal proteins, except RPL35A, for which Tat expression caused a marked decrease at the nucleolar level (0.29-fold). Similarly, several proteins involved in rRNA processing exhibited an overall increase in nucleolar accumulation upon Tat expression. These include human canonical members of the L7ae family together with members participating in Box C/D, H/ACA and U3 snoRNPs (Figure 4). Conversely, BOP1, a component of the PeBoW (Pescadillo Bop1 WDR12) complex essential for maturation of the large ribosomal subunit, was significantly depleted from the nucleolus of Jurkat TAP-Tat cells (0.81-fold) and this was confirmed by WB analysis (Figure 2) [93]. Nevertheless, the other PeBoW complex components, Pes1 (0.94-fold) and WDR12 (1.1-fold), were not affected by Tat expression. Of note, we did not detect change in the abundance of protein participating in rDNA transcription such as RNAPOLI, UBF.

#### Spliceosome

We identified and quantified in our dataset 55 proteins out of the 108 known spliceosomal proteins [94]. These proteins include the small nuclear ribonucleoproteins U1, U2 and U5, Sm D1, D2, D3, F and B, and the heterogeneous nuclear ribonucleoproteins. Our data suggested a distinct increase in the abundance of specific spliceosome complex proteins upon expression of HIV-1 Tat in Jurkat T-cells (Figure 3 and 4). The only three proteins that were significantly depleted from the nucleolus upon expression of HIV-1 Tat were RBMX (0.89-fold), HNRNPA2B1 (0.84-fold) and SNRPA (0.81-fold). Several investigations showed expression alteration in cellular splicing factors in HIV-1 infected cells [95], [96].

#### Molecular chaperones

We have identified several molecular chaperones, co-chaperones and other factors involved into proteostasis to be highly enriched in the nucleolus of T-cells upon Tat expression (Figure 3 and 4), many of which were previously characterised as part of the Tat nuclear interactome [63]. Several heat-shock proteins including DNAJs, specific HSP90, HSP70 and HSP40 isoforms and their co-factors were distinctively enriched in the nucleolar fraction of Jurkat cells expressing Tat (Figure 4). As shown by WB, while HSP90α and β are mostly cytoplasmic, Tat expression triggers their relocalisation to the nucleus and nucleolus, corroborating our proteomic quantitative approach (Figure 2). Similarly, heat-shock can cause the HSP90 and HSP70 to relocalise to the nucleolus [97], [98], [99], [100], [101]. In a recent study, Fassati’s group has shown that HSP90 is present at the HIV-1 promoter and may directly regulate viral gene expression [102]. We also observed the coordinated increased abundance of class I (GroEL and GroES) and class II (chaperonin containing TCP-1 (CTT)) chaperonin molecules (Figure 3 and 4) upon Tat expression.

#### Ubiquitin-proteasome pathway

The ubiquitin-proteasome pathway is the major proteolytic system of eukaryotic cells [103]. Importantly, the nuclear ubiquitin-proteasome pathway controls the supply of ribosomal proteins and is important to ribosome biogenesis [104], [105]. The 26S proteasome is composed of the 20S core particle (CP) and the 19S regulatory particle (RP). Alternatively, CP can associate with the 11S RP to form the immunoproteasome. All the quantified proteins in our study are part of the 19S regulatory complex and include PSMD2 (1.5-fold), PSMD3 (1.32-fold), PSMD11 (1.25-fold) and PSMD13 (0.72-fold), the only proteasome component significantly depleted from the nucleolus in the presence of Tat (Figure 4). Interestingly, Tat interacts with distinct subunits of the proteasome system, including the 19S, 20S and 11S subunits. The consequences of these interactions include the competition of Tat with 11S RP or 19S RP for binding to the 20S CP, which resulted in the inhibition of the 20S peptidase activity [106], [107], [108], [109], [110], [111]. Furthermore, Tat was shown to modify the proteasome composition and activity, which affects the generation of peptide antigens recognized by cytotoxic T-lymphocytes [112]. Importantly, a recent study demonstrated that in the absence of Tat, proteasome components are associated to the HIV-1 promoter and proteasome activity limits transcription [113]. Addition of Tat promoted the dissociation of the 19S subunit from the 20S proteasome, followed by the distinct enrichment of the 19S-like complex in nuclear extracts together with the Tat-mediated recruitment of the 19S subunits to the HIV-1 promoter, which facilitated its transcriptional elongation [113].

We also quantified UBA1 (1.36-fold), the E3 ubiquitin-protein ligase UHRF1 (1.13-fold), UBC (1-fold) and two Ubiquitin-specific-peptidases, USP30 (1.28-fold) and USP20 (0.06-fold) (Figure 4).

#### DNA replication and repair

Upon HIV-1 Tat expression, we observed the coordinated nucleolar enrichment of several cellular factors associated with DNA replication and repairs pathways (Figure 4).

Tat induced the coordinated enrichment of the miniature chromosome maintenance MCM2-7 complex (from 1.23- to 3.30-fold, respectively) [114]. MCM7, 6 and 3 were identified as part of the *in vitro* nuclear interactome of HIV-1 Tat [63]. The structural maintenance of chromosomes 2, SMC2, was enriched (1.35-fold) in the nucleolar fraction by Tat expression. SMC2 was identified as part of the *in vitro* nuclear interactome of HIV-1 Tat [63]. While replication factor C1 (RFC1) and RFC2 (1.31- and 1.28-fold respectively) displayed an increased fold change and RFC5/3 were not affected, RFC4 was severely depleted (0.69-fold) from the nucleolar fraction upon Tat expression [115]. RFC1 and RFC2 were identified as part of the *in vitro* nuclear interactome of HIV-1 Tat [63]. Tat induced the enrichment of XRCC6 (1.27-fold) and XRCC5 (1.36-fold) in the nucleolus, which are involved in the repair of non-homologous DNA end joining (NHEJ) [116]. XRCC6 associates with viral preintegration complexes containing HIV-1 Integrase and also interact with Tat and TAR [117], [118], [119]. Furthermore, in a ribozyme-based screen, XRCC5 (Ku80) knockdown decreased both retroviral integration and Tat-mediated transcription [120]. As part of the base excision repair (BER), we have identified a major apurinic/apyrimidinic endonuclease 1 **(**APEX1) (1.29-fold). Importantly, in a siRNA screen targeting DNA repair factors, APEX1 knockdown was found to inhibit HIV-1 infection by more 60% [121]. The high mobility group (HMG) protein, HMGA1 (1.30-fold), was enriched in the nucleolus following Tat expression [122]. HMGA1 interact with HIV-1 Integrase and is part of the HIV-1 pre-integration complex [123], [124]. Importantly, HMGA1 has been identified in a proteomic screen, as a cellular cofactor interacting with the HIV-1 5′leader [125].

#### Metabolism

Our proteomic data suggest that Tat induces perturbations in glycolysis, the pentose phosphate pathway, and nucleotide and amino acid biosynthesis (Figure 4 and Figure S7). Notably, in T cells expressing Tat, we detected co-ordinated changes in the abundance of proteins not previously known to be associated with Tat pathogenesis, which revealed unexpected connections with with glycolysis and the pentose phosphate pathway, including the following glycolitic enzymes, lactate dehydrogenase B (LDHB) (4.75-fold), Phosphofructokinase platelet (PFKP) (3.40-fold), pyruvate kinase M2 (PKM2) (1.59-fold), phosphoglycerate kinase 1 (PGK1)S (1.57-fold), glucose phosphate isomerase (GPI) (1.45-fold), triosephosphate isomerase 1 (TPI1) (1.37-fold), glyceraldehyde 3-phosphate dehydrogenase (GAPDH) (1.17-fold) and phosphoglyceric acid mutase (PGAM1) (0.89-fold) (Figure 4 and Figure S7). Briefly, GPI catalyzes the reversible isomerization of glucose-6-phosphate in fructose-6-phosphate. Subsequently, PFKP catalyzes the irreversible conversion of fructose-6-phosphate to fructose-1,6-bisphosphate and is a key regulatory enzyme in glycolysis. At the end of the glycolytic pathway, PKM2, in its tetrameric form, is known to generate ATP and pyruvate, while LDHB diverts the majority of the pyruvate to lactate production and regeneration of NAD+ in support to continued glycolysis, a phenomenon described for proliferative T-cells [126]. Of note, in highly proliferating cells, PKM2 can be found in its dimeric form and its activity is altered. This up-regulates the availibility of glucose intermediates, which are re-routed to the pentose phosphate and serine biosynthesis pathways for the production of biosynthetic precursors of nucleotides, phospholipids and amino acids. As part of the pentose phosphate pathway, we have characterised the significant enrichment of glucose-6-phosphate dehydrogenase (G6PD) (2.11-fold), which branches of the glycolysis pathway to generate NADPH, ribose-5-phosphate an important precursor for the synthesis of nucleotides. Consistent with this, we detected the coordinated increase in the abundance of enzymes which plays a central role in the synthesis of purines and pyrimidines. More specifically, IMPDH2 (1.66-fold), a rate-limiting enzyme at the branch point of purine nucleotide biosynthesis, leading to the generation of guanine nucleotides, phosphoribosyl pyrophosphate synthetase 2 (PRPS2) (1.41-fold), cytidine-5-prime-triphosphate synthetase (CTPS) (1.74-fold) which catalyses the conversion of UTP to CTP and the ribonucleotide reductase large subunit (RRM1) (1.56-fold). In parralel, we noted the increased abundance of the phosphoserine aminotransferase PSAT1 (1.90-fold), an enzyme implicated in serine biosynthesis, which has been linked with cell proliferation *in vitro*.

## Discussion

The host-virus interface is a fundamental aspect in defining the molecular pathogenesis of HIV-1 [127], [128], [129], [130], [131], [132], [133]. Indeed, with its limited repertoire of viral proteins, HIV-1 relies extensively on the host cell machinery for its replication. Several recent studies have capitalized on the recent advances in the “OMICS” technologies, and have revealed important insights into this finely tuned molecular dialogue [132], [134]. HIV-1 Tat is essential for viral replication and orchestrates HIV-1 gene expression. The viral regulatory protein is known to interact with an extensive array of cellular proteins and to modulate cellular gene expression and signaling pathway [135], [136]. We and others have employed system-level approaches to investigate Tat interplay with the host cell machinery, which have characterised HIV-1 Tat as a critical mediator of the host-viral interface [137], [138], [139], [140], [141], [142], [143], [144], [145], [146], [147], [148], [149]. Here, we have investigated the nucleolar proteins trafficking in response to HIV-1 Tat expression in T-cells, with the view to provide unique and novel insights on the role of proteins compartimentalisation by Tat in the fine-tuning of protein availability and function.

We have developed for this study, a cellular model using Jurkat T-cells stably expressing Tat fused in its N-ternminal to TAP-tag. Jurkat T-cells are robust and present the advantage to grow without stimulations and are easely transduced using retroviral gene delivery. Importantly, they have been widely employed to evaluate Tat-mediated pathogenesis using system-wide approaches and to analyse T-cell key cellular signaling pathways and functions [144], [150], [151], [152]. Indeed, we have found them particularly suited for prolongued *in vitro* culture in SILAC medium and subsequent isolation of their nucleolus followed by MS analysis, which requires up to 85 millions of cells. We fused Tat to the TAP tag to enable future downstream applications such as Tandem affinity purification or Chromatin IP analysis. Importantly, we have confirm that N-terminal TAP-tag did not interfere with Tat function nor its localisation in Jurkat cells, when compared to untagged-Tat. Of note, Tat subcellular distribution can vary according to the cell type employed. While Tat is known to accumulate in the nucleus and nucleolus in Jurkat cells and other transformed cell lines, in primary T-cells, Tat was described to primarily accumulate at the plasma membrane, while trafficking via the nucleus where it functions [32]. These differences remain to be characterised but could be related to different expression levels of transport factors in transformed cell lines versus primary cells, as recently described by Kuusisto *et al.* [39]. Furthermore, Stauber and Pavlakis have suggested that Tat nucleolar localisation could be the results of Tat overexpression [31]. Here, we have selected and employed a polyclonal population of Jurkat T-cells expressing Tat at different levels. We propose that this heterogeneity in Tat expression levels might reflect Tat stochastic expression described during viral replication [153].

Using a quantitative proteomic strategy based on an organellar approach, we quantified over 520 nucleolar proteins, including 49 proteins exhibiting a significant fold change. The extent to which the induced variations in the abundance of nucleolar proteins are biologically relevant and can affect cellular and/or viral processes remains to be determined. Nevertheless, the biological nature of the pathways and macromolecular complexes affected enable us to discuss their potential associations with HIV-1 pathogenesis.

HIV-1 Tat is expressed early following HIV-1 genome integration and mediates the shift to the viral production phase, associated with robust proviral gene expression, viral proteins assembly and ultimately, virions budding and release. In this context and based on our results, we propose that Tat could participate in shaping the intracellular environment and metabolic profile of T cells to favor host biosynthetic activities supporting robust virions production. Indeed, we observed the distinct nucleolar enrichment of ribosomal proteins and enzymes associated with ribosomal biogenesis, which could be indicative of an increase in protein synthesis. With the notable exeption of RPL35A nucleolar depletion, ribosomal proteins and enzymes associated with ribosomal biogenesis were in the top 20 most enriched nucleolar proteins (NHP2L1, RLP14, RPL17, RPL27, RPS2, RPL13). Furthermore, this effect appears to be specific to HIV-1 Tat since transcription inhibition by Actinomycin D resulted in the overall depletion of ribosomal proteins in the nucleolus [9]. Moreover, quantitative proteomics analysis of the nucleous in adenovirus-infected cells showed a mild decrease in ribosomal proteins [24]. Whether this reflect a shift in ribosome biogenesis and/or a change in the composition of the ribosomal subunits remains to be determined. Nevertheless, the adapted need for elevated ribosome production is intuitive for a system that needs to support the increased demand for new viral proteins synthesis. In parralel, we observed the concordant modulation of pathways regulating protein homeostasis. We noted the significant nucleolar accumulation of multiple molecular chaperones including the HSPs, the TCP-1 complex, and CANX/CALR molecules and the disrupted nucleolar abundance of proteins belonging to the ubiquitin-proteasome pathway, which controls the supply of ribosomal proteins [104], [105]. These observations further support previous studies describibing the modulation of the proteasomal activity by Tat, which affect the expression, assembly, and localization of specific subunits of the proteasomal complexes [106], [107], [108], [109], [110], [111], [113]. We also observed the concomitant depletion of CASP10 in the nucleolus of Jurkat TAP-Tat. It has been suggested that CASP10 could be targeted to the nucleolus to inhibit protein synthesis [154]. Interestingly, the presence and potential roles of molecular chaperones in the nucleolus have been highlighted by Banski et al, who elaborate on how the chaperone network could regulate ribosome biogenesis, cell signaling, and stress response [97], [155]. As viral production progresses into the late phase and cellular stress increases, nucleolar enrichment of molecular chaperones by Tat could not only enable adequat folding of newly synthetised viral proteins but could also promote tolerance of infected cells to stress and maintain cell viability.

Coincidentally, we observed the marked nucleolar enrichment of enzymes belonging to metabolic pathways including glycolysis, pentose phosphate, nucleotide and amino acid biosynthetic pathways. Similarly, these pathways are elevated in proliferative T-cells or in cancer cells following a metabolic shift to aerobic glycolysis, also known as the Warburg effect [156], [157], [158], [159]. There, glucose intermediates from the glycolysis pathway are not only commited to energy production and broke-down into pyruvate for the TCA cycle, but are redirected to alternative pathways, including the pentose phosphate pathway, and used as metabolic precursors to produce nucleotides, amino acids, acetyl CoA and NADPH for redox homeostasis. Consistently, we also noted the concomittant nucleolar enrichment of enzymes belonging to the nucleotide synthesis pathway, including IMPH2, a rate limiting enzyme known to control the pool of GTP. Similarly, we noted the nucleolar enrichment of PSAT1, an enzyme involved in serine and threonin metabolism, which is associated with cellular proliferation [160]. Collectively, we propose that by controlling protein homeostasis and metabolic pathways, Tat could meet both the energetic and biosynthetic demand of HIV-1 productive infection.

Of note, while nucleotide metabolism enzymes are associated with the nucleus, glycolysis takes place in the cytoplasm. Nevertheless, glycolytic enzymes have been detected in both the nuclear and nucleolar fractions by proteomic analyses [8], [161]. Furthermore glycolytic enzymes, such as PKM2, LDH, phosphoglycerate kinase, GAPDH, and aldolase, also have been reported to display nuclear localization and bind to DNA [162]. More specifically, PKM2 is known to associate with promoter and participate in the regulation of gene expression as a transcriptional coactivator [163].

HIV-1 Tat has previously been described as an immunoregulator and more specifically, has been reported both to inhibit or to promote TCR signaling [164]. We have observed the nucleolar enrichment by Tat of key proximal or downstream components of T-cell signaling pathways, including ZAP70, ILF3 and STAT3, which play crucial roles in T-cell development and activation. We had previously identified them as T-cell specific components of the nucleolus, and IF studies suggested that their association with the nucleolus could be regulated by specific conditions [165]. Our results further support that Tat could contribute to the dysregulation of TCR-derived signals and that the nucleolus could represent an important spatial link for TCR signaling molecules.

We observed the coordinated nucleolar enrichment of key components of the DNA replication, recombination and repair pathways by Tat. These include XRCC5 and XRCC6, HMGA1, APEX1, MCM2-7, SMC2, RFC1 and RFC2, while RFC4 was found to be significantly depleted. Interestingly, these cofactors have been associated with the efficiency of retroviral DNA integration into the host DNA or the integrity of integrated provirus [166]. Whether the increased abundance of these factors within the nucleolus could be associated with their potential participation in the integration and maintenance of provirus gene integrity, remains to be determined.

The mechanisms of Tat-mediated segregation and compartimentalisation of proteins in or out of the nucleolus may depend on factor(s) inherent for each protein and the nature of their relationship with Tat, since subcellular fractionation combined with WB analysis showed that the pattern and extent of subcellular redistribution between proteins varied. We could observe cases where Tat upregulated the expression of proteins which resulted in a general increase of theses proteins throughout the cellular compartments including the nucleolus (DDX3, TNPO1). Alternatively, Tat could trigger the nucleolar translocation of proteins directly from the cytoplasm or the nucleoplasm (pRb). Additionally, we observed cytoplasmic proteins redistributed to both the nucleoplasm and nucleolus upon Tat expression (STAT3, ZAP70 and HSP90). Finally, we also noted protein depletion in the nucleolar fraction accompanied by an increase in the nucleoplasm (SSRP1). It remains difficult at this stage, to appreciate whether the accumulation of specific proteins would result in their activation or inhibition by sequestering them away from their site of action. Conversely, the depletion of a protein from the nucleolus could either result in the down-regulation of its activity in this location or could be the result of its mobilization from its storage site, the nucleolus, to the nucleoplasm or cytoplasm where it can perform its function. Remarkably, we identified several known HIV-1 Tat partners involved in HIV-1 pathogenesis, which suggests that Tat could physically modulate their nucleolar targeting or their recruitment to specific site in the nucleoplasm or cytoplasm. Tat could also promote post-translational modifications, which could mediate the targeting of specific proteins to the nucleolus. This is exemplified by the following enriched proteins, pRb, PP1 and STAT3, for which phosphorylation is induced by Tat. Importantly, their phosphorylation status determines their subcellular distribution, thus providing a potential mechanism for their redistribution by Tat. Moreover, our data indicates that serine/threonine kinases (CK2 α’) and phosphatases (PP1) were significantly enriched in the nucleolar fractions of Jurkat TAP-Tat. These enzymes account for the majority of the phosphorylation/dephosphorylation activity in the nucleolus and can act as regulators of nucleolar protein trafficking. In addition, Tat significantly decreased the levels of SUMO-2 in the nucleolus. Similarly, SUMO-mediated post-translational modifications are known to modulate nucleolar protein localization [104]. Given the potential importance of post-translational modifications, including phosphorylation in the Tat-mediated change of abundance of nucleolar proteins, a more targeted proteomic approach such as the enrichment for phosphopetides, would extend the resolution of our screening approach.

The control of protein turnover is also an important mean to modulate the abundance of nucleolar proteins. Ribosomal proteins are degraded by the Ubiquitin-Proteasome pathway to ensure their abundance matches up with rRNA transcription levels. Conversely, heat shock proteins HSP90s protect them from degradation. Interestingly, our data showing that Tat modulation the abundance proteins associated with the Ubiquitin-proteasome and heat-shock pathway. This could contribute to the observed enrichment of ribosomal proteins by Tat. Nevertheless, we cannot exclude that the increased abundance of ribosomal proteins in the nucleolus could be the result of Tat-mediated prevention of their export to the cytoplasm. Interestingly, using a different cellular system, a *drosophila melanogaster* Tat transgenic strain, Ponti *et al*, analysed the effects of Tat on ribosome biogenesis, following 3 days heat shock treatment to induce Tat expression under the control of the hsp70 promoter [167]. Following Tat expression, they observed a defect in pre-rRNA processing associated with a decrease in the level of 80S ribosomes [167]. Nevertheless, the different cellular system employed combined with the 3 days heat-shock induction make their results difficult to compare with ours.

### Conclusions

While previous system-level studies have monitored the effects of HIV-1 Tat expression on T cells, to our knowledge, we have presented here the first proteomic analysis of dynamic composition of the nucleolus in response to HIV-1 Tat expression. Using quantitative proteomics, we have underlined the changes in abundance of specific nucleolar proteins and have highlighted the extensive and coordinated nucleolar reorganization in response to Tat constitutive expression. Our findings underscore that Tat expressing T-cells exhibit a unique nucleolar proteomic profile, which may reflect a viral strategy to facilitate the progression to robust viral production. Importantly, we noted the functional relationship of nucleolar proteins of our dataset with HIV-1 pathogenesis and HIV-1 Tat in particular. This further increases our confidence in our experimental strategy and suggests a role for Tat in the spatial control and subcellular compartimentaliation of these cellular cofactors. Ultimatly, our study provides new insights on the importance of Tat in the cross talk between nucleolar functions and viral pathogenesis. Importantly, we have also identified changes in nucleolar protein abundance that were not previously associated with HIV-1 pathogenesis, including proteins associated with metabolic pathways, which provide new potential targets and cellular pathways for therapeutic intervention.

## Materials and Methods

### Cell Culture

Jurkat T-cells, clone E6.1 (ATCC), Jurkat NTAP-Tat and Jurkat NTAP were maintained in RPMI-1640 medium supplemented with 10% (v/v) foetal bovine serum (Gibco, EU approved), and antibiotics. Phoenix-GP cells (G.P. Nolan; www.stanford.edu/group/nolan/), were maintained in DMEM medium supplemented with 10% (v/v) foetal bovine serum (GIBCO, EU approved). Cells were counted using Scepter™ 2.0 Cell Counter (Millipore).

### Establishment of Stable Cell Lines Expression HIV-1 Tat

The sequence of HIV-1 Tat (HIV-1 HXB2, 86 amino acids) was sub-cloned into pENTR 2B vector (Invitrogen, A10463). Using the Gateway technology (Invitrogen), we introduced the HIV-1 Tat sequence into the plasmid pCeMM-NTAP(GS)-Gw [168]. Phoenix cells (G.P. Nolan; www.stanford.edu/group/nolan/), were transfected using Fugene 6 (Roche) with 5 µg of the plasmid NTAP-Tat or NTAP and 3 µg of the pMDG-VSVG. Viral supernatants were collected after 48 h, filtered and used to transduce the Jurkat cell lines. The construct is termed NTAP-Tat, the empty vector was termed NTAP. Using retroviral gene delivery, we stably transduced Jurkat cells (clone E6.1 (ATCC)). The positive clones named Jurkat NTAP-Tat and Jurkat NTAP were sorted to enrich the population of cells expressing GFP using the BC MoFlo XDP cell sorter (Beckman Coulter).

### Electrophoresis and Western-Blotting Analysis

Sub-cellular fractions (10 µg) were resolved by SDS-PAGE and transferred onto BioTrace PVDF membranes (Pall corporation). The following primary antibodies were used: α-Tubulin (Sc 5286), C23 (Sc 6013), and Fibrillarin (Sc 25397) were from Santa Cruz Biotechnology, and PARP (AM30) from Calbiochem, mouse anti-ZAP 70 (05–253, Millipore), rabbit anti-STAT3 (06–596, Millipore), rabbit anti-ILF3 (ab92355, Abcam), rabbit anti-HSP90 beta (ab32568, Abcam), mouse anti-ADAR1 (ab88574, Abcam), rabbit anti-HDAC1 (ab19845, Abcam), rabbit anti-SSRP1 (ab21584, Abcam) rabbit anti-BOP1 (ab86982, Abcam), mouse anti-KpNB1 (ab10303, Abcam), rabbit anti-HIV-1 Tat (ab43014, Abcam), rabbit anti-CK2A (ab10466, Abcam), rabbit anti-DDX3X (ab37160, Abcam), mouse anti-TNPO1 (ab2811, Abcam), mouse anti-HSP90A (CA1023, MERCK), and rabbit-anti RB1 (sc-102, Santa Cruz).The following secondary antibodies were used ECL: Anti-mouse IgG and ECL Anti-rabbit IgG (GE Healthcare), and Donkey anti-goat IgG (Sc 2020) (Santa Cruz Biotechnology).

### SILAC Labeling and Nucleolar Isolation

For SILAC analysis SILAC-RPMI R0K0 and SILAC-RPMI R6K6 (Dundee cells) media supplemented with 10% dialyzed FBS (GIBCO, 26400-036) were used. The Jurkat cells expressing NTAP-Tat and NTAP were serially passaged and grown for five doublings to ensure full incorporation of the labelled amino acids. Cells viability was checked with Trypan Blue (0.4% solution, SIGMA) and further confirmed using PI staining and FACS analysis. Cells were mixed to the ratio 1∶1 to obtain 140×10^6^ cells. Nucleoli were isolated from the mixed cell population as previously described in Jarboui *et al.*, [165].

### In-Solution Digestion and Nano LC-MS/MS

Nucleolar extracts (100 µg) were resuspended in 50 mM ammonium bicarbonate and in solution trypsin digested as previously described in Jarboui *et al.* [165]. Sample was run on a Thermo Scientific LTQ ORBITRAP XL mass spectrometer connected to an Eksigent NANO LC.1DPLUS chromatography system incorporating an auto-sampler. Sample was loaded onto a Biobasic C18 PicofritTM column (100 mm length, 75 mm ID) and was separated by an increasing acetonitrile gradient, using a 142 min reverse phase gradient (0–40% acetonitrile for 110 min) at a flow rate of 300 nL min-1. The mass spectrometer was operated in positive ion mode with a capillary temperature of 200°C, a capillary voltage of 46V, a tube lens voltage of 140V and with a potential of 1800 V applied to the frit. All data was acquired with the mass spectrometer operating in automatic data dependent switching mode. A high resolution MS scan was performed using the Orbitrap to select the 5 most intense ions prior to MS/MS analysis using the Ion trap.

### SILAC Incorporation Efficiency

The incorporation efficiency of labelled amino-acids was determined by analysing the peptides identified in isolated nucleoli from cell population maintained in “Heavy” medium as described in [169]. Our analysis showed that we had an incorporation efficiency >95% (data not shown).

**MS/MS Data Analysis and Proteins Quantification**

The MS/MS spectra were searched for peptides identification and quantification using the MaxQuant software [170] (version 1.1.1.36), the Human IPI Database (version 3.83) and the Andromeda search engine associated to MaxQuant [171]. Standard settings were used for MaxQuant with the Acetyl (Protein N-term) as variable modification and Carbamidomethyl (Cys) as fixed modification, 2 missed cleavage were allowed, except that the filtering of labelled amino acids was prohibited. Initial mass deviation of precursor ion and fragment ions were 7 ppm and 0.5 Da, respectively. Each protein ratio was calculated as the intensity-weighted average of the individual peptides ratios. Proteins were identified with the minimum of one peptide with a false discovery rate less than 1%.

### Gene Ontology and Data Analysis

Gene ontology, KEGG pathway and Pfam terms were extracted from UNIPROT entries using Perseus, a software from the MaxQuant Data analysis package (http://www.maxquant.org ), and the ToppGene suite tools [54].

### Luciferase Assay

The Jurkat NTAP-Tat and Jurkat NTAP were transfected using the Amaxa electroporation system (Amaxa biosystem) with the pGL3 (pGL3-LTR) (Promega) as recommended by Amaxa Biosystem. Dual-luciferase assays (Promega) were performed according to the manufacturer’s instructions. Luciferase activity was measured and normalized against the total amount of proteins as quantified by the BCA protein quantification kit (Pierce, Thermo Scientific).

### Immunofluorescence

To preserve their original shape, we performed immunostaining of Jurkat cells in suspension. Cells were fixed in 2% PFA for 10 min at RT, permeabilised in 0.5% Triton X-100 for 15 min at RT and blocked with 5% FCS. Cells were incubated with the rabbit HIV-1 Tat antibody (ab43014, Abcam) followed by the secondary antibody anti-Rabbit alexa fluor 647 (A-21246, Invitrogen). Cells were allowed to attach to Cell-Tak (BD) coated Silanised Slides (DaoCytomation), and stained with DAPI. Images were captured with a Carl Zeiss Confocal Microscope equipped with a Plan-Apochromat 63X/1.4 oil DIC objective.

### Data Deposition in Proteomecommons

The proteomics RAW Data file from the mass spectrometry analysis was deposited to the Tranche repository(https://proteomecommons.org/tranche/) [172]. The file can be accessed and downloaded using the following hash key:

(R3O5SV5Z6HvWqrBNDhp21tXFetluDWYxvwMIfUh6e1kMgarauCSq4dlNcxeUvFOHDEzLeDcg4X5Y8reSb6MUA6wM1kIAAAAAAAAB/w =  = ).

## Supporting Information

Figure S1 **Comparaison of the activity of TAP-Tat versus untagged Tat in stably transfected Jurkat T-cells.** Jurkat NTAP-Tat, Jurkat-tat and Jurkat cells were transfected with 5 µg of pGL3-LTR plasmid. These results are the mean (+/−SD) of two independents experiment performed in triplicate. HIV-1 LTR luciferase reporter gene assay confirmed that the NTAP-Tat is functionally active.
(TIFF)

Figure S2 **Sub-cellular localisation of HIV-1 Tat in stably transfected Jurkat-tat and Jurkat TAP-Tat cell lines.** Jurkat-tat and Jurkat TAP-Tat cells were stained for fibrillarin (green), HIV-1 Tat (red), and DAPI (grey contrast). (Bar: 10 µm). Images were captured using a Nikon Eclipse Ti E Spinning Disk microscope (Andor), equipped with a 100X/1.3 N.A oil objective and 488 nm, 595 nm and 405 nm laser lines. A z-stack of the nucleus was obtained with slices of 0.2 µm. Subsequently, deconvolution was performed using AutoquantX2 (Media Cybernetics).
(TIF)

Figure S3 **Expression and subcellular distribution of Tat in Jurkat-tat cells using Western-Blot analysis.** HIV-1 Tat (15 kDa) was detected using anti-HIV-1 Tat antibody (ab43014, Abcam). (Fractions: WC: whole cells, C: Cytoplasmic, N: Nuclear, Np: Nucleoplasmic and No: Nucleolar).
(TIF)

Figure S4 **Cell cycle and cell proliferation analysis of Jurkat NTAP and NTAP-Tat. A.** Jurkat NTAP-Tat and Jurkat NTAP were labeled with 5 uM Cell Proliferation Dye eFluor® 670, then cultured for 7 days and analyzed by FACS. **B.** Cell cycle FACS analysis of Jurkat NTAP-Tat (red) and Jurkat NTAP (green) following PI staining.
(TIF)

Figure S5 **Topology analysis and clustering coefficient distribution of the T-cell nucleolar network upon expression of HIV-1 Tat.** A. Topology analysis using Centiscape plugin showed that the node degree distribution of the main network follows a power low. B. The nucleolar network of cells expressing HIV-1 Tat exhibit a hierarchical architecture as the distribution of the clustering coefficient versus the node degree in a logarithmic plot, follows a straight-line slope. The highly connected ribosomal proteins form a distinct group on the upper right corner of the plot.
(TIF)

Figure S6 **HIV-1 Tat centrality analysis.** HIV-1 Tat is a central protein in the network, as most of its centralities were above the network average.
(TIF)

Figure S7 **Distinct sets of metabolic enzymes are enriched in the nucleolus of Jurkat T-cell expressing Tat.** The glycolytic and pentose phosphate pathways are presented with the corresponding metabolic enzymes quantified highlighted in red (enriched) and green (depleted).
(TIFF)

Table S1 **Lists of all the peptides identified and quantified in our analysis and used to calculate the proteins SILAC ratios.** The table contains 536 proteins identified from 2471 peptides.
(XLSX)

Table S2 **List of the proteins previously described to interact with HIV-1 Tat and identified in our quantitative analysis of the nucleoli of Jurkat T-cells expressing NTAP-Tat.** Data gathered from the HIV-1 human protein interaction database. (www.ncbi.nlm.nih.gov/RefSeq/HIVInteractions/)
(DOCX)

Materials and Methods S1 **Description of the methods employed to examine cell cycle, cell viability and cell proliferation analysis.**
(DOCX)
