# Supplementary figures and images for: Nucleolar Protein Trafficking in Response to HIV-1 Tat: Rewiring the Nucleolus
Source: PLoS One. 2012 Nov 15;7(11):e48702. doi: 10.1371/journal.pone.0048702 (PMC3499507; doi:10.1371/journal.pone.0048702)

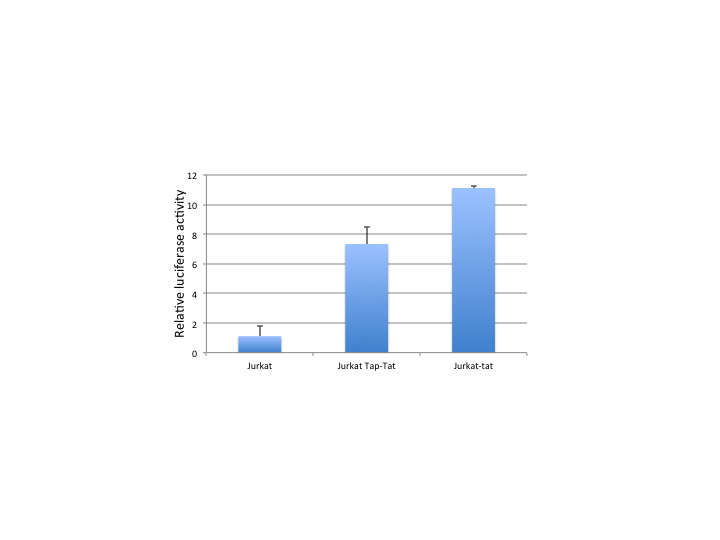

Supplement: Figure S1 — Comparaison of the activity of TAP-Tat versus untagged Tat in stably transfected Jurkat T-cells. Jurkat NTAP-Tat, Jurkat-tat and Jurkat cells were transfected with 5 µg of pGL3-LTR plasmid. These results are the mean (+/−SD) of two independents experiment performed in triplicate. HIV-1 LTR luciferase reporter gene assay confirmed that the NTAP-Tat is functionally active. (TIFF) [file pone.0048702.s001.tiff]

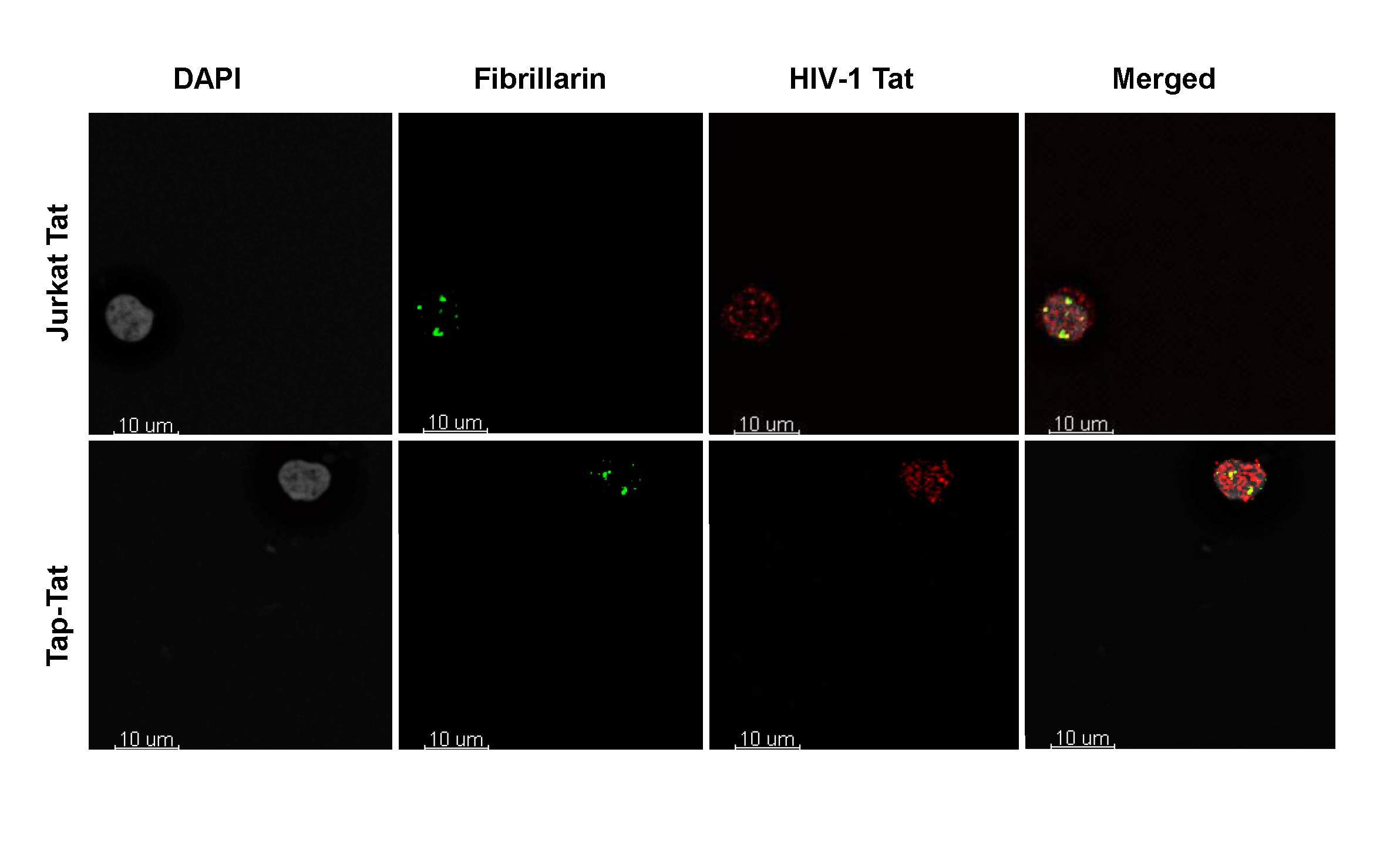

Supplement: Figure S2 — Sub-cellular localisation of HIV-1 Tat in stably transfected Jurkat-tat and Jurkat TAP-Tat cell lines. Jurkat-tat and Jurkat TAP-Tat cells were stained for fibrillarin (green), HIV-1 Tat (red), and DAPI (grey contrast). (Bar: 10 µm). Images were captured using a Nikon Eclipse Ti E Spinning Disk microscope (Andor), equipped with a 100X/1.3 N.A oil objective and 488 nm, 595 nm and 405 nm laser lines. A z-stack of the nucleus was obtained with slices of 0.2 µm. Subsequently, deconvolution was performed using AutoquantX2 (Media Cybernetics). (TIF) [file pone.0048702.s002.tif]

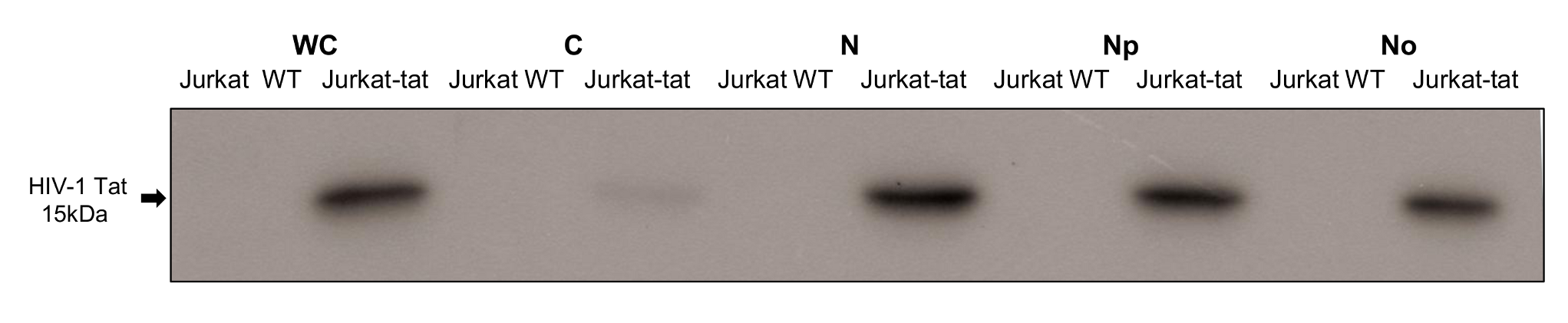

Supplement: Figure S3 — Expression and subcellular distribution of Tat in Jurkat-tat cells using Western-Blot analysis. HIV-1 Tat (15 kDa) was detected using anti-HIV-1 Tat antibody (ab43014, Abcam). (Fractions: WC: whole cells, C: Cytoplasmic, N: Nuclear, Np: Nucleoplasmic and No: Nucleolar). (TIF) [file pone.0048702.s003.tif]

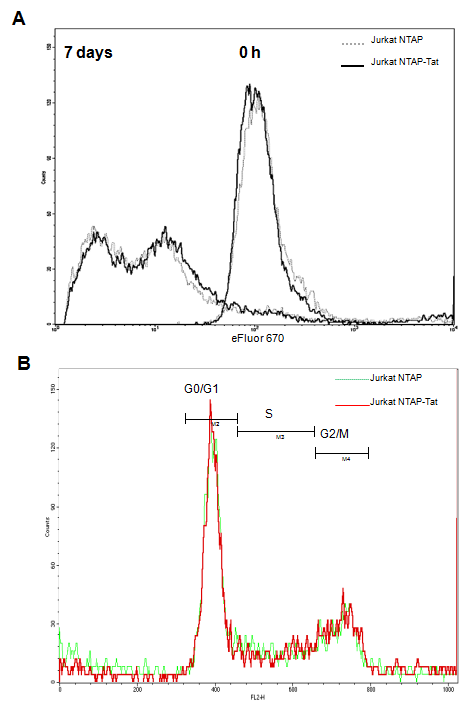

Supplement: Figure S4 — Cell cycle and cell proliferation analysis of Jurkat NTAP and NTAP-Tat. A. Jurkat NTAP-Tat and Jurkat NTAP were labeled with 5 uM Cell Proliferation Dye eFluor® 670, then cultured for 7 days and analyzed by FACS. B. Cell cycle FACS analysis of Jurkat NTAP-Tat (red) and Jurkat NTAP (green) following PI staining. (TIF) [file pone.0048702.s004.tif]

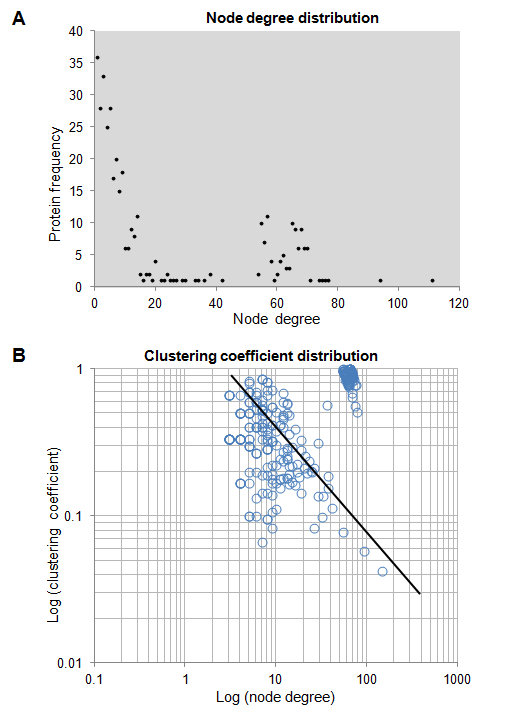

Supplement: Figure S5 — Topology analysis and clustering coefficient distribution of the T-cell nucleolar network upon expression of HIV-1 Tat. A. Topology analysis using Centiscape plugin showed that the node degree distribution of the main network follows a power low. B. The nucleolar network of cells expressing HIV-1 Tat exhibit a hierarchical architecture as the distribution of the clustering coefficient versus the node degree in a logarithmic plot, follows a straight-line slope. The highly connected ribosomal proteins form a distinct group on the upper right corner of the plot. (TIF) [file pone.0048702.s005.tif]

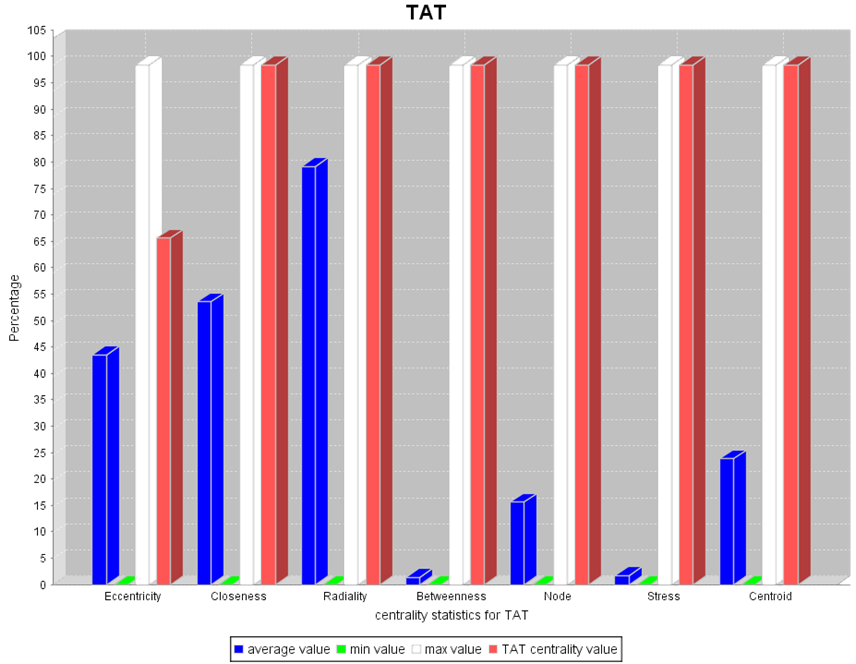

Supplement: Figure S6 — HIV-1 Tat centrality analysis. HIV-1 Tat is a central protein in the network, as most of its centralities were above the network average. (TIF) [file pone.0048702.s006.tif]

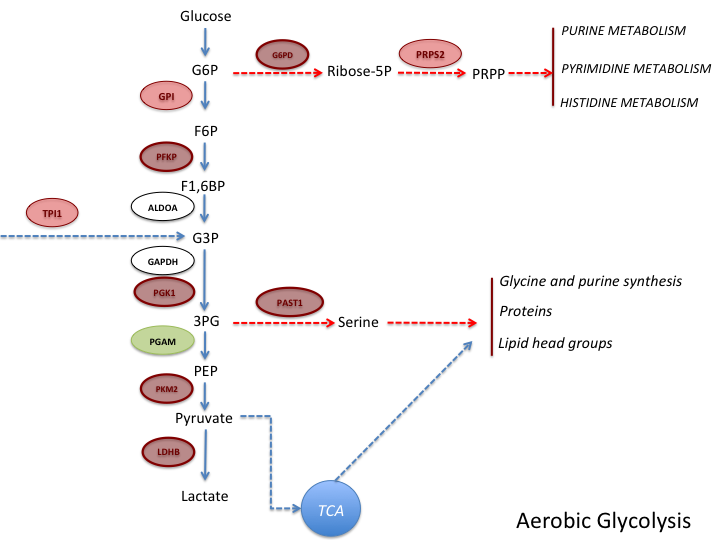

Supplement: Figure S7 — Distinct sets of metabolic enzymes are enriched in the nucleolus of Jurkat T-cell expressing Tat. The glycolytic and pentose phosphate pathways are presented with the corresponding metabolic enzymes quantified highlighted in red (enriched) and green (depleted). (TIFF) [file pone.0048702.s007.tiff]
