# Supplementary material for: Nucleolar Protein Trafficking in Response to HIV-1 Tat: Rewiring the Nucleolus
Source: PLoS One. 2012 Nov 15;7(11):e48702. doi: 10.1371/journal.pone.0048702 (PMC3499507; doi:10.1371/journal.pone.0048702)
Supplement: Table S2 — List of the proteins previously described to interact with HIV-1 Tat and identified in our quantitative analysis of the nucleoli of Jurkat T-cells expressing NTAP-Tat. Data gathered from the HIV-1 human protein interaction database. (www.ncbi.nlm.nih.gov/RefSeq/HIVInteractions/) (DOCX) [file pone.0048702.s009.docx]

| **Gene Name** | **Gene Description** | **SILACRatios** | **Log2 Ratios** |
| --- | --- | --- | --- |
| **G6PD** | glucose-6-phosphate dehydrogenase | 2.11 | 1.08 |
| **STAT3** | signal transducer and activator of transcription 3 (acute-phase response factor) | 1.88 | 0.91 |
| **MYH9** | myosin, heavy chain 9, non-muscle | 1.63 | 0.71 |
| **MCM6** | minichromosome maintenance complex component 6 | 1.56 | 0.64 |
| **PPP1CA** | protein phosphatase 1, catalytic subunit, alpha isoform | 1.53 | 0.61 |
| **CBX3** | similar to chromobox homolog 3 | 1.51 | 0.59 |
| **PSMD2** | proteasome (prosome, macropain) 26S subunit, non-ATPase, 2 | 1.50 | 0.59 |
| **RB1** | retinoblastoma 1 | 1.44 | 0.53 |
| **PHB** | prohibitin | 1.42 | 0.51 |
| **EIF2S3** | eukaryotic translation initiation factor 2, subunit 3 gamma, 52kDa | 1.41 | 0.50 |
| **MATR3** | matrin 3 | 1.39 | 0.48 |
| **HSPA5** | hypothetical gene supported by AF216292 | 1.39 | 0.47 |
| **YBX1** | Y box binding protein 1 | 1.38 | 0.47 |
| **NSUN2** | NOL1/NOP2/Sun domain family, member 2 | 1.37 | 0.46 |
| **RANBP1** | similar to RAN binding protein 1 | 1.37 | 0.46 |
| **DNAJB1** | DnaJ (Hsp40) homolog, subfamily B, member 1 | 1.36 | 0.45 |
| **UBA1** | ubiquitin-like modifier activating enzyme 1 | 1.36 | 0.45 |
| **HSP90AA1** | heat shock protein 90kDa alpha (cytosolic), class A member 2 | 1.36 | 0.44 |
| **XRCC5** | X-ray repair complementing defective repair in Chinese hamster cells 5 (double-strand-break rejoining) | 1.36 | 0.44 |
| **POLR2H** | polymerase (RNA) II (DNA directed) polypeptide H | 1.35 | 0.44 |
| **SMC2** | structural maintenance of chromosomes 2 | 1.35 | 0.43 |
| **CCT4** | chaperonin containing TCP1, subunit 4 (delta) | 1.35 | 0.43 |
| **DHX15** | DEAH (Asp-Glu-Ala-His) box polypeptide 15 | 1.33 | 0.42 |
| **PSMD3** | proteasome (prosome, macropain) 26S subunit, non-ATPase, 3 | 1.33 | 0.41 |
| **EEF1D** | eukaryotic translation elongation factor 1 delta (guanine nucleotide exchange protein) | 1.31 | 0.39 |
| **RHOA** | ras homolog gene family, member A | 1.31 | 0.39 |
| **RFC1** | replication factor C (activator 1) 1, 145kDa | 1.31 | 0.38 |
| **LEF1** | lymphoid enhancer-binding factor 1 | 1.30 | 0.38 |
| **TBL3** | transducin (beta)-like 3 | 1.30 | 0.38 |
| **PPP2R1A** | protein phosphatase 2 (formerly 2A), regulatory subunit A, alpha isoform | 1.29 | 0.37 |
| **RFC2** | replication factor C (activator 1) 2, 40kDa | 1.28 | 0.35 |
| **RPSA** | ribosomal protein SA pseudogene 9 | 1.28 | 0.35 |
| **TNPO1** | transportin 1 | 1.28 | 0.35 |
| **KPNA2** | karyopherin alpha 2 (RAG cohort 1, importin alpha 1) | 1.27 | 0.35 |
| **XRCC6** | X-ray repair complementing defective repair in Chinese hamster cells 6 | 1.27 | 0.35 |
| **HSPA9** | heat shock 70kDa protein 9 (mortalin) | 1.27 | 0.35 |
| **RAC2** | ras-related C3 botulinum toxin substrate 2 (rho family, small GTP binding protein Rac2) | 1.26 | 0.33 |
| **PSMD11** | proteasome (prosome, macropain) 26S subunit, non-ATPase, 11 | 1.25 | 0.32 |
| **ZAP70** | zeta-chain (TCR) associated protein kinase 70kDa | 1.24 | 0.31 |
| **NUP93** | nucleoporin 93kDa | 1.24 | 0.31 |
| **MCM2** | minichromosome maintenance complex component 2 | 1.23 | 0.30 |
| **HSPA8** | heat shock 70kDa protein 8 | 1.23 | 0.29 |
| **PHB2** | prohibitin 2 | 1.22 | 0.29 |
| **ENO1** | enolase 1, (alpha) | 1.22 | 0.29 |
| **DDX3X** | DEAD (Asp-Glu-Ala-Asp) box polypeptide 3, X-linked | 1.21 | 0.28 |
| **CFL1** | cofilin 1 (non-muscle) | 1.21 | 0.27 |
| **SF3B1** | splicing factor 3b, subunit 1, 155kDa | 1.20 | 0.27 |
| **GNB2L1** | guanine nucleotide binding protein (G protein), beta polypeptide 2-like 1 | 1.20 | 0.26 |
| **BAZ1B** | bromodomain adjacent to zinc finger domain, 1B | 1.20 | 0.26 |
| **EEF1A1** | eukaryotic translation elongation factor 1 alpha-like 7 | 1.20 | 0.26 |
| **KPNB1** | karyopherin (importin) beta 1 | 1.18 | 0.23 |
| **GAPDH** | glyceraldehyde-3-phosphate dehydrogenase-like 6 | 1.17 | 0.23 |
| **SSB** | Sjogren syndrome antigen B (autoantigen La) | 1.17 | 0.22 |
| **RPL3** | ribosomal protein L3 | 1.16 | 0.22 |
| **ACTA2** | actin, alpha 2, smooth muscle, aorta | 1.15 | 0.20 |
| **C1QBP** | complement component 1, q subcomponent binding protein | 1.14 | 0.19 |
| **EIF6** | eukaryotic translation initiation factor 6 | 1.14 | 0.19 |
| **TAF15** | TAF15 RNA polymerase II, TATA box binding protein (TBP)-associated factor, 68kDa | 1.13 | 0.17 |
| **PDCD11** | programmed cell death 11 | 1.12 | 0.17 |
| **HNRNPD** | heterogeneous nuclear ribonucleoprotein D (AU-rich element RNA binding protein 1, 37kDa) | 1.11 | 0.16 |
| **RFC5** | replication factor C (activator 1) 5, 36.5kDa | 1.11 | 0.16 |
| **PRKDC** | similar to protein kinase, DNA-activated, catalytic polypeptide | 1.10 | 0.14 |
| **LMNB2** | lamin B2 | 1.09 | 0.13 |
| **HDAC2** | histone deacetylase 2 | 1.09 | 0.12 |
| **RFC3** | replication factor C (activator 1) 3, 38kDa | 1.08 | 0.12 |
| **SNRPA1** | small nuclear ribonucleoprotein polypeptide A' | 1.08 | 0.11 |
| **H2AFY** | H2A histone family, member Y | 1.08 | 0.11 |
| **SUB1** | SUB1 homolog (S. cerevisiae) | 1.06 | 0.09 |
| **IFI16** | interferon, gamma-inducible protein 16 | 1.06 | 0.09 |
| **RPA1** | replication protein A1, 70kDa | 1.06 | 0.08 |
| **PCNA** | proliferating cell nuclear antigen | 1.05 | 0.08 |
| **RBBP4** | hypothetical LOC642954 | 1.05 | 0.07 |
| **PRKCG** | protein kinase C, gamma | 1.05 | 0.07 |
| **SNRPD1** | small nuclear ribonucleoprotein D1 polypeptide 16kDa | 1.05 | 0.07 |
| **UBTF** | upstream binding transcription factor, RNA polymerase I | 1.04 | 0.06 |
| **CBX5** | chromobox homolog 5 (HP1 alpha homolog, Drosophila) | 1.04 | 0.05 |
| **HDAC1** | histone deacetylase 1 | 1.03 | 0.05 |
| **FBL** | fibrillarin | 1.03 | 0.05 |
| **LMNB1** | lamin B1 | 1.03 | 0.04 |
| **DHX9** | DEAH (Asp-Glu-Ala-His) box polypeptide 9 | 1.02 | 0.03 |
| **NUMA1** | nuclear mitotic apparatus protein 1 | 1.02 | 0.03 |
| **SNRPB** | small nuclear ribonucleoprotein polypeptides B and B1 | 1.01 | 0.02 |
| **TARDBP** | TAR DNA binding protein | 1.01 | 0.01 |
| **ILF2** | interleukin enhancer binding factor 2, 45kDa | 1.00 | 0.01 |
| **PARP1** | poly (ADP-ribose) polymerase 1 | 1.00 | 0.00 |
| **H2AFV** | H2A histone family, member V | 1.00 | 0.00 |
| **PTBP1** | polypyrimidine tract binding protein 1 | 0.99 | -0.01 |
| **TOP2A** | topoisomerase (DNA) II alpha 170kDa | 0.99 | -0.01 |
| **SNRPD3** | small nuclear ribonucleoprotein D3 polypeptide 18kDa | 0.99 | -0.02 |
| **HIST2H2AB** | histone cluster 2, H2ab | 0.98 | -0.03 |
| **SUPT16H** | suppressor of Ty 16 homolog (S. cerevisiae) | 0.97 | -0.05 |
| **H2AFJ** | H2A histone family, member J | 0.97 | -0.05 |
| **HIST1H3A** | histone cluster 1, H3j | 0.96 | -0.05 |
| **NPM1** | nucleophosmin 1 (nucleolar phosphoprotein B23, numatrin) pseudogene 21 | 0.96 | -0.06 |
| **SNRPD2** | small nuclear ribonucleoprotein D2 polypeptide 16.5kDa | 0.96 | -0.06 |
| **HIST1H2BJ** | histone cluster 1, H2bj | 0.95 | -0.08 |
| **HIST2H4A** | histone cluster 1, H4l | 0.95 | -0.08 |
| **ILF3** | interleukin enhancer binding factor 3, 90kDa | 0.94 | -0.08 |
| **PES1** | pescadillo homolog 1, containing BRCT domain (zebrafish) | 0.94 | -0.09 |
| **HIST1H2BD** | histone cluster 1, H2bd | 0.94 | -0.09 |
| **HIST2H2AA3** | histone cluster 2, H2aa3 | 0.92 | -0.11 |
| **HIST1H2AE** | histone cluster 1, H2ae | 0.92 | -0.12 |
| **TMPO** | thymopoietin | 0.92 | -0.12 |
| **SMARCA4** | SWI/SNF related, matrix associated, actin dependent regulator of chromatin, subfamily a, member 4 | 0.91 | -0.13 |
| **SSRP1** | structure specific recognition protein 1 | 0.89 | -0.17 |
| **ADAR** | adenosine deaminase, RNA-specific | 0.78 | -0.36 |
| **PSMD13** | proteasome (prosome, macropain) 26S subunit, non-ATPase, 13 | 0.72 | -0.47 |
| **RFC4** | replication factor C (activator 1) 4, 37kDa | 0.69 | -0.53 |
