# Supplementary material for: Nucleolar Protein Trafficking in Response to HIV-1 Tat: Rewiring the Nucleolus
Source: PLoS One. 2012 Nov 15;7(11):e48702. doi: 10.1371/journal.pone.0048702 (PMC3499507; doi:10.1371/journal.pone.0048702)
Supplement: Materials and Methods S1 — Description of the methods employed to examine cell cycle, cell viability and cell proliferation analysis. (DOCX) [file pone.0048702.s010.docx]

**Material and methods S1**

**Cell cycle, cell viability and cell proliferation analysis**

Cells were stained with 0.4% trypan blue dye (Sigma T8154) and counted using phase contrast microscopy. For the cell cycle analysis, Jurkat cells were fixed in 4% paraformaldehyde solution and stained with propidium iodide as described previously [[1](#_ENREF_173)]. Cell viability was determined by trypan blue exclusion. Cell proliferation was performed using the Dye eFluor 670 cell labelling (eBioscience, 65-0840) as recommended by the manufacturer. FACS analyses were performed using the FACSCalibur flow cytometer (BD).

**Supporting References**

1. Li X, Gong J, Feldman E, Seiter K, Traganos F, et al. (1994) Apoptotic cell death during treatment of leukemias. Leukemia & lymphoma 13 Suppl 1: 65-70.
